# ROS-Induced DNA Damage Associates with Abundance of Mitochondrial DNA in White Blood Cells of the Untreated Schizophrenic Patients

**DOI:** 10.1155/2018/8587475

**Published:** 2018-02-25

**Authors:** I. V. Chestkov, E. M. Jestkova, E. S. Ershova, V. G. Golimbet, T. V. Lezheiko, N. Yu Kolesina, O. A. Dolgikh, V. L. Izhevskaya, G. P. Kostyuk, S. I. Kutsev, N. N. Veiko, S. V. Kostyuk

**Affiliations:** ^1^Research Centre for Medical Genetics (RCMG), Moscow 115478, Russia; ^2^N. A. Alexeev Clinical Psychiatric Hospital №1 of Moscow Healthcare Department, Moscow 115447, Russia; ^3^V. A. Negovsky Research Institute of General Reanimatology, Federal Clinical Research Center of Reanimatology and Rehabilitogy, Moscow 107031, Russia; ^4^Mental Health Research Center, Moscow 115522, Russia

## Abstract

**Objective:**

The aim of this study was (1) to examine the leukocyte mtDNA copy number (*CN*) in unmedicated (SZ (m−)) and medicated (SZ (m+)) male patients with paranoid schizophrenia (SZ) in comparison with the healthy male controls (HC) and (2) to compare the leukocyte mtDNA *CN* with the content of an oxidation marker 8-oxodG in lymphocytes of the SZ (m−) patients.

**Methods:**

We evaluated leukocyte mtDNA *CN* of 110 subjects with SZ in comparison with 60 male HC by the method qPCR (ratio mtDNA/nDNA (gene B2M) was detected). SZ patients were divided into two subgroups. The patients of the subgroups SZ (m+) (*N* = 55) were treated with standard antipsychotic medications in the hospital. The patients of the subgroup SZ (m−) (*N* = 55) were not treated before venous blood was sampled. To evaluate oxidative DNA damage, we quantified the levels of 8-oxodG in lymphocytes (flow cytometry) of SZ (m−) patients (*N* = 55) and HC (*N* = 30).

**Results:**

The leukocyte mtDNA *CN* showed no significant difference in SZ (m+) patients and HC. The mtDNA *CN* in the unmedicated subgroup SZ (m−) was significantly higher than that in the SZ (m+) subgroup or in HC group. The level of 8-oxodG in the subgroup SZ (m−) was significantly higher than that in HC group.

**Conclusion:**

The leukocytes of the unmedicated SZ male patients with acute psychosis contain more mtDNA than the leukocytes of the male SZ patients treated with antipsychotic medications or the healthy controls. MtDNA content positively correlates with the level of 8-oxodG in the unmedicated SZ patients.

## 1. Introduction

Approximately 1% of the world's population suffer from schizophrenia (SZ). Schizophrenia is a highly heritable neuropsychiatric disorder of complex genetic etiology. Mitochondrion (mt) is a cellular organelle involved in the regulation of a variety of complex cellular processes. Mitochondria, the cell energy source, have a crucial role in intracellular calcium homeostasis, producing ROS and activating the apoptotic pathway. MtDNA copy number (*CN*) variation has been suggested as a sensitive index of cellular oxidative stress, inflammation, and mitochondrial dysfunction [1, 2].

The accumulating morphological, genetic, and imaging data delineates mitochondrial multifaceted dysfunction as a pathological factor in schizophrenia [3]. Several studies have evaluated the influence of schizophrenia on mtDNA copy number. Results are controversial to each other. Some authors show lower mtDNA copy number in brain tissues and peripheral lymphocytes of schizophrenia patients compared with healthy controls (HC) [4–7]. Some studies reported no anomalous mtDNA copy number in the tissues of schizophrenia patients [8, 9]. The reason for the observed contradictions may be the lack of sufficient data on the effect of antipsychotic therapy on mtDNA copy number. It is known that administration of antipsychotics involved mitochondrial functions, especially OXPHOS. The antipsychotics inhibit the mitochondrial respiratory chain [3]. However, there are few studies on the impact of antipsychotics on mtDNA content in schizophrenia patients. Li et al. recently had showed that antipsychotic risperidone causes a decrease in the number of mtDNA copies in leukocytes of patients with the first-episode antipsychotic-naïve schizophrenia [4]. Thus, the first objective of our work was to determine the effects of the standard antipsychotic therapy on the number of mtDNA copies in the cells of male patients with paranoid schizophrenia.

Systemic oxidative stress is associated with schizophrenia. Elevated reactive oxygen species (ROS) levels and declined antioxidant statuses have been reported in the brain and peripheral tissues of the patients with schizophrenia [10–12]. However, it is known that oxidative stress in the body is associated with the changes in mtDNA copy number. For example, oxidative stress caused by environmental exposure, for example, ionizing radiation, induces ROS-caused increase of the amount of mtDNA in the human and animal cells and organisms [13–15].

Thus, we can assume that a high level of ROS in the body of some schizophrenia patients should correlate with an increased mtDNA copy number. So, the second objective of our work was to test this hypothesis. We quantified mtDNA content and the levels of oxidative stress marker 8-oxodG in the white blood cells of male patients with untreated paranoid schizophrenia.

## 2. Methods

### 2.1. Subjects

This investigation was approved by the Regional Ethics Committee of RCMG. It was carried out in accordance with the latest version of the Declaration of Helsinki. One hundred ten male paranoid SZ patients with acute psychotic disorders were recruited from the general psychiatric units for treatment of acute forms of mental disorders (Psychiatric Hospital #14 of Moscow City Health Department and Mental Health Research Center, Moscow). All participants signed an informed written consent to participate in this investigation. Age of the patients: 38 ± 13 (19–62). Clinical symptoms have been measured using the positive and negative syndromes scale (PANSS) (Kay et al. [16]), a widespread instrument proven to be valid and suitable for evaluation of positive, negative, and general psychopathological items. It includes three subscales, totally 30 items: 7 for positive symptoms, 7 for negative, and 16 for general psychopathological ones. Each symptom has 7 ratings (1—absent, 2—questionable, 3—mild, 4—moderate, 5—severe, 6—markedly severe, and 7—extremely severe). The PANSS interviews, completed by a trained researcher, were conducted one week before the patient's discharge from the hospital. Patients were diagnosed with paranoid schizophrenia (F20.00 and F20.01 in the International Classification of Diseases 10th Revision (ICD-10)) using structured mini international neuropsychiatric interview (MINI). Diagnoses were also confirmed pursuant to DSM-IV criteria. The paranoid SZ duration was less than 3 years (*n* = 24, 22%), 3–10 years (*n* = 20, 18%), and more than 10 years (*n* = 66, 60%). No subjects suffered from any relevant disease.

SZ patients were divided into groups: SZ (m+) (*N* = 55) and SZ (m−) (*N* = 55). The patients in group SZ (m+) received standard antipsychotic therapy in the hospital for at least six weeks before the venous blood was sampled. They were clinically stable and their medications had not changed for at least one month. For the treatment of the acute disorders, standard antipsychotics were used: haloperidol, chlorpromazine, clozapine, risperidone, quetiapine, and olanzapine. Most of the patients of the group SZ (m−) refused to take antipsychotics in the home despite of the chronic course of the disease. These patients did not take antipsychotics for 3 to 8 months until a new hospitalization with acute psychosis. Some patients of the group SZ (m−) (*N* = 12, 21.8%) were diagnosed as the first-episode SZ and were never treated with antipsychotic medications.

From the SZ (m−) patients, the venous blood was collected on the day of hospitalization before starting any antipsychotic therapy. 20 mL of blood was collected from a peripheral vein of the subject using a syringe flushed with heparin under strict aseptic conditions. The control group consisted of 60 healthy males with no history of any psychiatric disorder, correlating with the patient group by age (37 ± 12 (17–73)) and by their smoking habits.

### 2.2. Isolating of DNA from the Blood

The leukocytes were isolated from 5 mL of blood by the method of Boyum [17]. 5 mL of the solution containing 2% sodium lauryl sarcosylate, 0.04 M EDTA, and 150 *μ*g/mL RNAse A (Sigma, USA) was added to the fresh leucocytes for 45 min (37°C) and then was treated with proteinase K (200 *μ*g/mL, Promega, USA) for 24 h at 37°C. The lysate samples were extracted with an equal volume of phenol, phenol/chloroform/isoamyl alcohol (25 : 24 : 1), and chloroform/isoamyl alcohol (24 : 1), respectively. DNA was precipitated by adding 1/10 volume of 3 M sodium acetate (pH 5.2) and 2.5 volume of ice-cold ethanol. For the extraction procedure, only freshly distilled solvents were used. Phenol was stabilized with 8-hydroxyquinoline. Finally, the DNA was collected by centrifugation at 10,000*g* for 15 min at 4°C, washed with 70% ethanol (*v*/*v*), dried, and dissolved in water. The quantitation of the purified genomic DNA is performed using the PicoGreen dsDNA quantitation reagent from Molecular Probes (Invitrogen, CA, USA). The assay displays a linear correlation between dsDNA quantity and fluorescence over a wide range. The DNA concentration of the samples is calculated according to a DNA standard curve. We use EnSpire equipment (Finland) with the following parameters: excitation and emission wavelengths 488 and 528 nm, respectively.

### 2.3. Quantitative Real-Time PCR (qPCR)

Serial qPCR assay was established using the StepOnePlus (Applied Biosystems). QPCR efficiencies for ten samples were determined by serially diluted genomic DNA. MtDNA copy number determinations in the other samples were obtained using a calculated average efficiency (E + 1) of 2.0 ± 0.05 for gene B2M and 1.95 ± 0.03 for mtDNA. Each reaction contained 10 *μ*L 2хSYBR Premix Ex Taq (PerfectRealTime™, Takara Bio), 2 *μ*L primers (10 *μ*M), and 8 *μ*L of DNA (5 ng/*μ*L) for a final volume of 20 *μ*L. All reactions were performed in duplicates. PCR conditions were 6 min at 95°C initial denaturation, followed by 40 cycles of 30 s of denaturation at 95°C, 15 s of primer annealing at 60°C, and 10 s at 72°C of extension. The presence of unspecific amplicons was excluded by melting curve analysis and gel electrophoresis. The following primers (Malik et al. [18]) were used (Sintol, Russia):

Human mitochondrial genome NC_012920 (D-loop)

hmito (65) F CTTCTGGCCACAGCACTTAAAC; R GCTGGTGTTAGGGTTCTTTGTTTT

Human B2M (accession number M17987)

hB2M (95) F GCTGGGTAGCTCTAAACAATGTATTCA; R CCATGTACTAACAAATGTCTAAAATGG

### 2.4. Flow Cytometry Analysis (FCA) of 8-oxodG

Method is described in detail earlier [19]. Briefly, lymphocytes were isolated from 15 mL of the fresh blood. To fix the cells, 2% paraformaldehyde (Sigma) was used (37°C, 10 min). Cells were permeabilized with 0.1% Triton X-100 (Sigma) in PBS (15 min, 25°C). Cells were stained with FITC-8-oxodG Abcam antibody (1 *μ*g/mL, 4 h, 4°C). We stained a portion of the cells with secondary FITC-conjugated antibodies to determine the level of the background fluorescence. Cells were analyzed at CyFlow Space (Partec, Germany). Primary data are presented as median values of the signal FL1 (8-oxodG). Index 8-oxodG = (I−Ib)/Ib, where I and Ib are the experienced and the background signal.

### 2.5. Statistics

All reported results were reproduced at least two times as independent biological replicates.

The significance of the observed differences was analyzed using the nonparametric Mann–Whitney *U*-tests. All *p* values were two-sided and considered statistically significant at *α* < 0.05.

For the statistical analysis was used Professional software StatPlus2007 (http://www.analystsoft.com/).

## 3. Results

### 3.1. Quantification of mtDNA in Leukocytes of HC and SZ Groups

**Figure 1:**
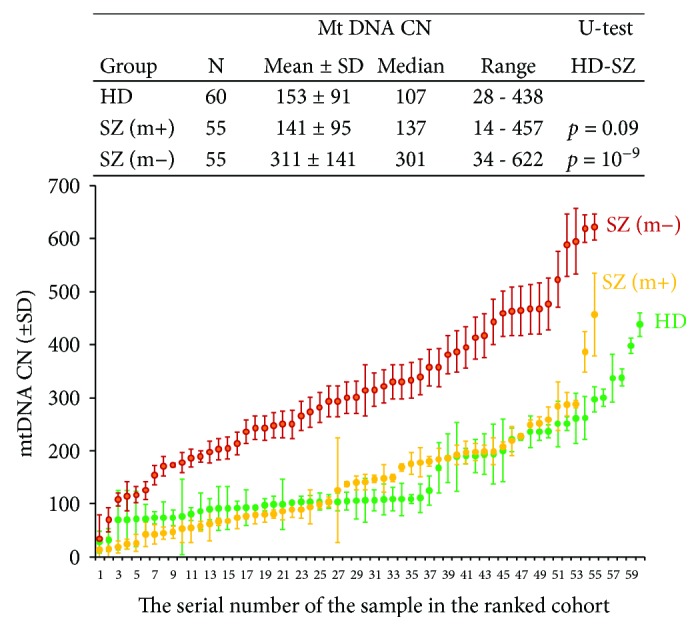
Leukocytes from unmedicated SZ patients with acute psychosis are enriched with mtDNA. Graph—Quantification of mtDNA in leukocytes of HC (green), SZ (m−) (red), and SZ (m+) (yellow) groups. The data in each group is ranked by the value of mtDNA *CN*. The mean and standard deviation for each DNA sample is given (the data of two independent experiments, three measurements in each experiment, *n* = 6). Table contains statistical data.

The mtDNA *CN* in the leukocytes of SZ patients and HC detected by qPCR (mtDNA to nDNA (gene B2M) ratio was measured) is given in Figure 1. Comparison of the samples by the Mann–Whitney test is given in the table in Figure 1. In 12 patients of 55 (21.8%) from SZ (m−) group and only in 1 patients of 55 (1.7%) from SZ (m+) group, a considerable increase in mtDNA *CN* compared to the HC was found (Figure 1). Median mtDNA *CN* for the leukocytes of the unmedicated SZ (m−) patients was 2.8 or 2.2 times higher than the median mtDNA *CN* for the leukocytes of the medicated SZ (m+) or HC. The samples of HC and SZ (m+) did not differ among themselves in the content of mtDNA in leukocytes.

We did not find any significant correlation between the clinical symptoms (indexes of the scale PANSS) and the number of copies of mtDNA for the medicated SZ patients (*k* < 0.2; *p* > 0.1, linear regression method).

### 3.2. Quantification of 8-oxodG in Lymphocytes of HC and SZ (m−) Groups

**Figure 2:**
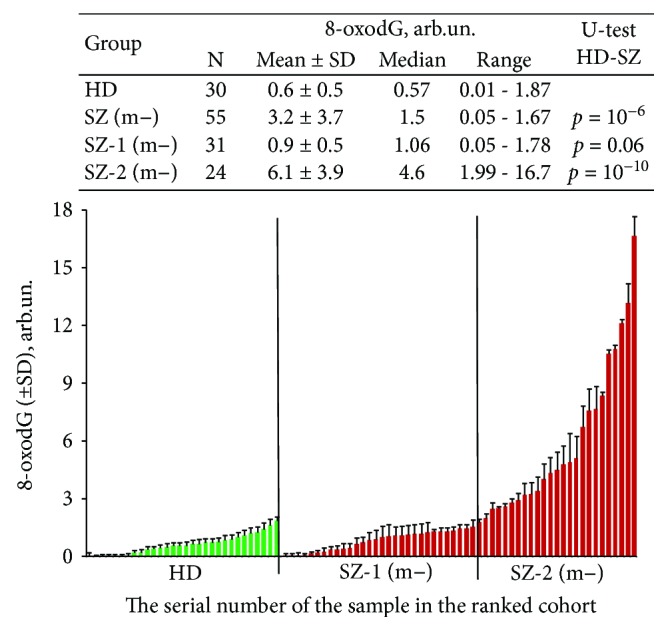
Lymphocytes from the unmedicated SZ patients with acute psychosis are enriched with 8-oxodG. Flow cytometry analysis of 8-oxodG. Index 8-oxodG (arb.un) is the ratio of the difference between the experienced and the background signal to the magnitude of the background signal. Graph—Quantification of 8-oxodG in lymphocytes of HC (green) and SZ (m−) (red) groups. The data in each group is ranked by the value of 8-oxodG. The mean and standard deviation for each cell sample is given (the data of three independent experiments, two measurements in each experiment, *n* = 6). Table contains statistical data.

FCA was employed in order to evaluate 8-oxodG content in the lymphocytes (Figure 2). Staining of the fixed cells with 8-oxodG antibodies was performed [19]. Figure 2 shows the mean values of the signal against the baseline values (8-oxodG index) for the groups HC and SZ (m−) (see table in Figure 2 for descriptive statistics). The 8-oxodG content in SZ (m−) lymphocytes was higher than in the control group (*p* = 10^−6^). The patients from the SZ (m−) group were divided into subgroups: SZ-1(m−) (*N* = 31) with normal 8-oxodG content and SZ-2 (m−) (*N* = 24) with increased 8-oxodG content compared to control. These groups differ in the level of cellular DNA oxidation (*p* = 10^−10^) (Figure 2).

### 3.3. The Dependence of mtDNA CN on 8-oxodG in HC and SZ (m−) Groups

**Figure 3:**
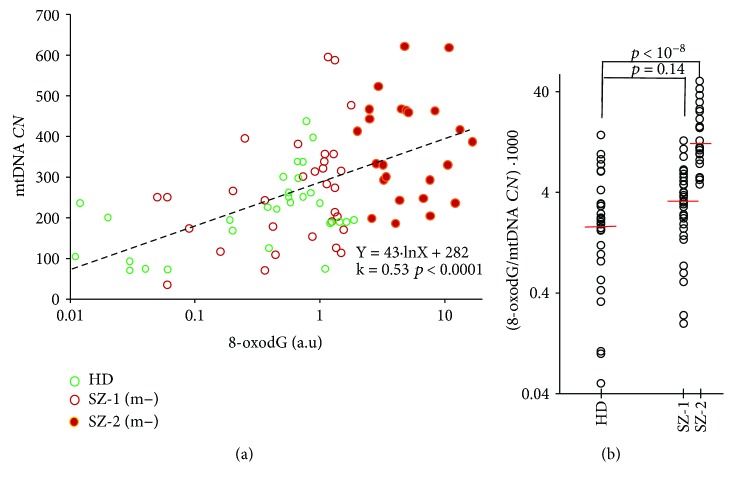
(a) The dependence of mtDNA *CN* on 8-oxodG (logarithmic scale) for unmedicated SZ (m−) and control groups. The SZ (m−) group was divided into two subgroups (SZ-1 and SZ-2). SZ-1 contains patients with normal content of 8-oxodG. Healthy donors belong to the same category. SZ-2 contains patients with high level of 8-oxodG. The graph shows the trend line and the linear regression equation. (b) Ratio 8-oxodG/mtDNA *CN* (logarithmic scale) is defined for three groups. Short lines denote medians. The comparison ratio 8-oxodG/mtDNA *CN* in the groups by the *U*-test is given.

Figure 3(a) presents dependence of mtDNA *CN* in the leukocytes on logarithm of the 8-oxodG content in the lymphocytes for control and SZ (m−) groups. In the control group (*N* = 30), correlation between the two parameters was found (*k* = 0.47; *p* < 0.01). In the total SZ (m−) group (*N* = 55), linear correlation between lg (8-oxodG) and mtDNA *CN* content was also found (*k* = 0.44; *p* < 0.001). The correlation between the analyzed parameters was detected for the total sample of healthy and schizophrenic people (*N* = 85, *k* = 0.53, *p* < 0.0001). Thus, the greater the level of DNA oxidation in the human cells, the greater the content of mtDNA.

The ratio (8-oxodG/mtDNA *CN*) indicates the amount of 8-oxodG per one copy of mtDNA (Figure 3(b)). This ratio was significantly higher in the SZ-2 group than in the group SZ-1 or HC. For 11 patients of 55, this index reached very high values in comparison with the rest of the SZ (m−) sample. All these patients were sick with schizophrenia for more than 10 years and smoked more than 40 cigarettes a day. However, for the whole group SZ (m−), we did not find a pronounced dependence of the indices 8-oxodG, mtDNA *CN*, and (8-oxodG/mtDNA *CN*) on the duration of schizophrenia or on the intensity of smoking.

We also analyzed the indices 8-oxodG, mtDNA *CN*, and (8-oxodG/mtDNA *CN*) in connection with the 29 indices of standard biochemical analysis of venous blood. We did not find any significant correlations.

## 4. Discussion

SZ is increasingly considered as a systemic disorder, which is associated with biochemical disturbances not only in the CNS/brain cells [20]. In recent years, there has been an intensive search for blood-based biomarkers for SZ [21]. The study of white blood cells can give an understanding of how the whole body responds to disturbances that eventually lead to the disorders of a brain function.

### 4.1. Abundance of the mtDNA CN in Leucocytes of the Unmedicated SZ Patients

The first finding of this study is higher mtDNA *CN* in the unmedicated SZ patients than in the medicated patients and the healthy controls (Figure 1). We have shown that acute psychosis of the unmedicated SZ (m−) patients is accompanied by a significant increase in the mtDNA *CN* in leucocytes (Figure 1). The leukocytes of SZ (m+) patients with the antipsychotic medication contain the amount of mtDNA comparable to the control. There are few studies on the impact of antipsychotics on mtDNA content in SZ patients. Li et al. recently also demonstrated that antipsychotic risperidone causes a decrease in the mtDNA *CN* in leukocytes of patients with the first-episode antipsychotic-naïve schizophrenia [4].

The reason for reduction of mtDNA *CN* in the medicated SZ patients remains unknown. The patients in this study were treated with various typical and atypical antipsychotics. In a state of acute psychosis, patients usually were treated with typical antipsychotics. After improving the mental state of the patient, he was treated with atypical antipsychotics. This fact did not allow us to form large enough groups to analyze the effect of specific antipsychotics on mtDNA *CN*. Recent studies have suggested that some antipsychotic drugs have a useful therapeutic effect by reducing oxidative stress in schizophrenic patients [22, 23]. Perhaps these drugs cause a decrease in the mtDNA *CN* through the antioxidant effects. It is also possible that antipsychotics change mitophagy in the cells of SZ patients. Mitophagy is a highly specific quality control process which eliminates dysfunctional mitochondria and promotes mitochondrial turnover [24]. Further studies are needed to understand the reasons for reducing the number of copies of mtDNA in the treated patients.

### 4.2. Abundance of mtDNA in the SZ (m−) Patients Associates with Oxidative DNA Damage

The lymphocytes of some unmedicated SZ patients contain more 8-oxodG than the lymphocytes of healthy controls (Figure 2). 8-oxodG represents major oxidative DNA damage products. An elevated level of 8-oxodG in the blood and urine has been reported in SZ patients [25, 26]. Postmortem studies have revealed a higher level of 8-oxodG in the brains of SZ patients compared to the controls [27, 28]. So our findings are consistent with those of other author who showed a high level of ROS in the SZ patient's body.

An internal source of ROS is present in the body of a patient with SZ [10–12]. The main cause of oxidative stress in schizophrenia has not yet been found. Different mechanisms of oxidative stress in schizophrenia have been postulated: dopamine metabolism, NO metabolism, abnormalities in the mitochondrial electron transport chain patients with schizophrenia, mutations in the genes responsible for maintaining the required level of ROS in cells, and so on. However, regardless of the cause, the result is important: in the cells of some SZ patients, the level of ROS is significantly increased. We can compare this situation with the effect on the cells of an external source of ionizing radiation, which induces oxidative stress in human. This model is attractive because it is well studied [13–15, 29–32]. Moreover, some authors hypothesized that ionizing radiation may be an environmental trigger that can actualize a predisposition to human schizophrenia or indeed cause schizophrenia-like disorders [33, 34]. Technogenic catastrophes (Chernobyl and Fukushima), atomic explosions (Japan and the region of the Kazakhstan nuclear weapon testing area), and areas with high natural background radiation (in India) several times increase the risk of schizophrenia in the population. The recent SZ model study suggests that irradiation of rats in adulthood caused behavioral abnormalities relevant to schizophrenia [35].

**Figure 4:**
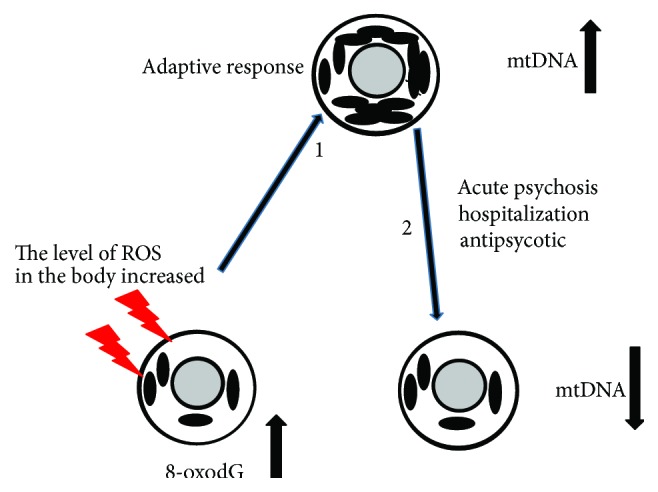
The scheme illustrates the choice of the parameters in the work. Schizophrenia is associated with elevated levels of ROS. Oxidative stress is known to cause the DNA damage. 8-oxodG is a marker of oxidative stress. (1) In response to increased levels of ROS and DNA damage, the cell increases the number of mitochondria. This response of the SZ patient's cells is similar to the response of healthy control's cells to oxidative stress induced by ionizing radiation [15]. (2) Hospitalization of the SZ patient and its treatment with antipsychotics leads to a decrease in the number of mtDNA in leukocytes.

In response to the oxidative stress, the cells of the unmedicated SZ patient (Figure 4) as well as cells of exposed to ionizing radiation human increase the amount of mtDNA. There is extensive literature on the increase in the number of mtDNA copies in response to ionizing radiation [13–15]. It is interesting to note that the effect of increasing mtDNA *CN* in untreated SZ-2 (m−) patients (average 2.8-fold compared with the control) is comparable to the effect of increasing mtDNA *CN* in leukemia patients undergoing total body irradiation therapy with a dose of 9 Gy (average 2.3-fold [36]). This may indicate that the levels of oxidative stress in some schizophrenia patient are comparable to the level of oxidative stress induced by sufficiently high doses of ionizing radiation.

It is currently unclear functional significance of an increased mitochondrial content induced by ROS. Some authors suggest that ROS could result in an adaptive response through enhanced production of mitochondria [15]. In conditions of oxidative stress, the transcriptional and replication machinery of mitochondrial biogenesis will be upregulated resulting in increased mitochondrial biogenesis [15, 37, 38]. Some authors proposed that the stress response of cells in terms of mitochondrial copy numbers could be a key for the life or death of the cell under oxidative stress [4, 39–41]. According to this hypothesis, an increase in mtDNA content may precede mitochondrial dysfunction as an adaptive response and could therefore be a predictive biomarker.

The above-mentioned hypothesis is confirmed by the data obtained in this study. Analyzing the oxidation marker 8-oxodG in SZ patients, we found that the unmedicated SZ patients can be distinctively divided into subgroups with different levels of 8-oxodG (Figure 2). MtDNA *CN* was much higher in the subgroup SZ-2 (m−) with a high level of 8-oxodG (Figure 3). On the one hand, the correlation between lg (8-oxodG) and mtDNA *CN* (Figure 3(a)) for HC and SZ(m−) can be explained by the preferential oxidation of mtDNA compared to nuclear DNA. MtDNA is highly susceptible to oxidative damage due to lack of protective histones and limited DNA repair capacity [42]. It can be assumed that the more copies of oxidized mtDNA in the cell, the higher the index 8-oxodG. However, very high values of the ratio 8-oxodG/mtDNA *CN* (Figure 3(b)) may indicate significant damage of the nuclear DNA. In the future, it is interesting to compare the ratio of 8-oxodG/mtDNA *CN* with the content of 8-oxodG in mtDNA and nuclear DNA using the PCR method.

It is still unclear whether peripheral findings reflect alterations in the brain of SZ patients at the time of an acute psychotic disorder. Nevertheless, we can suggest that enhanced copy number of mtDNA in the brain under the middle oxidative stress can contribute to abnormal behavioral symptoms in paranoid SZ. To answer the question about the role of an increased amount of mtDNA in patients with unmedicated acute psychosis, further studies are required.

## Additional Points

*Highlights*. (i) Unmedicated SZ men contain more mtDNA in leukocytes than medicated ones and control. (ii) Unmedicated SZ men contain more 8-oxodG in lymphocytes than healthy control. (iii) MtDNA content positively correlates with the level of 8-oxodG in unmedicated SZ patients.
